# Performance of Finnish biobanks in nationwide pulmonary carcinoid tumour research

**DOI:** 10.1007/s00428-019-02625-6

**Published:** 2019-08-05

**Authors:** Tiina Vesterinen, Kaisa Salmenkivi, Harri Mustonen, Teijo Kuopio, Elisa Lappi-Blanco, Timo Paavonen, Paula Vainio, Aija Knuuttila, Olli Carpén, Caj Haglund, Johanna Arola

**Affiliations:** 1 HUSLAB, Department of Pathology, University of Helsinki and Helsinki University Hospital, Haartmaninkatu 3, Helsinki, Finland; 2 Institute for Molecular Medicine Finland (FIMM), HiLIFE, University of Helsinki, Tukholmankatu 8, Helsinki, Finland; 3 Department of Surgery, University of Helsinki and Helsinki University Hospital, Haartmaninkatu 4, Helsinki, Finland; 4 Department of Biological and Environmental Science, University of Jyväskylä, Survontie 9, Jyväskylä, Finland; 5 Department of Pathology, Central Finland Health Care District, Keskussairaalantie 19, Jyväskylä, Finland; 6 Department of Pathology, Center for Cancer Research and Translational Medicine, Oulu University Hospital and University of Oulu, Aapistie 5, Oulu, Finland; 7 Department of Pathology, Fimlab Laboratories and Department of Medicine and Health Technology, Tampere University, Arvo Ylpön katu 34, Tampere, Finland; 8 Department of Pathology, University of Turku and Turku University Hospital, Kiinamyllynkatu 4-8, Turku, Finland; 9 Department of Pulmonary Medicine, Heart and Lung Center, and Cancer Center, University of Helsinki and Helsinki University Hospital, Haartmaninkatu 4, Helsinki, Finland; 10 Research Program in Systems Oncology, University of Helsinki, Haartmaninkatu 8, Helsinki, Finland; 11 Translational Cancer Medicine Research Program, Faculty of Medicine, University of Helsinki, Haartmaninkatu 8, Helsinki, Finland

**Keywords:** Biobank, Rare cancer, Pulmonary carcinoid, Prognosis

## Abstract

Finnish hospital-integrated biobanks administer millions of formalin-fixed paraffin-embedded tissue samples collected within the clinical diagnostics. According to the Finnish Biobank Act, these samples can be coupled with patients’ clinical follow-up data and the data retrieved from national health registries. We collected a nationwide pulmonary carcinoid tumour series from Finnish biobanks to study prognostic factors as well as to explore how the number of tumours found in the Finnish biobanks corresponds to the number of tumours registered by the Finnish Cancer Registry (FCR). Finnish biobanks identified 88% of the tumours registered by the FCR and were able to deliver 63%. The main reasons for lacking samples were paucity of resected primary tumour tissue, incompatible primary diagnosis, and the absence of tissue blocks in the archives. The main bottleneck in the sample application process was retrieving patient data. Altogether, we received 224 tumour samples with appropriate patient data and identified six prognostic factors for shorter disease-specific survival: age over 56 years at the time of diagnosis, tumour size over 2.5 cm, atypical histology, Ki-67 proliferation index higher than 2.5%, hilar/mediastinal lymph node involvement at the time of diagnosis, and the presence of metastatic disease. In conclusion, the Finnish biobank infrastructure offers excellent opportunities for tissue-based research. However, to be able to develop the biobank operations further, involving more medical knowledge in the sample and data acquisition process is a necessity. Also, when working with tissue samples collected over decades, histological expertise is essential for re-evaluation and re-classification of the samples.

## Introduction

Human biological samples with associated clinical data are a fundamental resource for advancing medical research. At the European level, hundreds of biobanks aim to assist researchers by providing biological materials [1]. At their best, biobanks can be valuable tools for preclinical, clinical, and translational studies aiming to advance our understanding of human health and disease. Within Europe, the BBMRI-ERIC research infrastructure consortium coordinates biobanking-related activities across countries. However, the legislation, organization of biobanks, and their capabilities across countries vary considerably.

Finland enacted specific legislation for biobanking in September 2013 providing the legal framework for the collection, storage, and usage of samples and related clinical data for biomedical research [2]. Between 2014 and 2018, 10 biobanks were established and started sample collection. Six of the biobanks are university or central hospital integrated collecting mainly blood and tissue samples from their patients. The rest are national biobanks who collect and administer samples, e.g. from healthy blood donors or for the needs of population-based health studies. Since every Finn has a unique personal identity number, combining biological samples with data from multiple national or local health registers is straightforward.

Informed consent is the primary justification for processing the samples and data. However, this consent can be broad and cover several unspecified future research purposes in which samples and data can be utilized. In addition, the Finnish Biobank Act also affords a pathway for transferring the clinical samples, stored within the healthcare system at the time the law entered into force, to biobanks without consent. In this setting, the approval is given by a regional ethics committee, and the individuals concerned are notified and given the possibility to opt out. Major Finnish clinical pathology laboratories have utilized this opportunity and transferred their sample archives into local hospital-integrated biobanks.

A cancer is typically defined as ‘common’ or ‘rare’ based on its incidence. While no universally recognized cutoff exists, the RARECARE–Surveillance of Rare Cancers in Europe working group defines a rare cancer to occur at a frequency of 6 cases per 100 000 individuals per year [3]. At the European level, 24% of all new cancer diagnoses each year are considered rare [4].

One of the rare cancer forms is pulmonary carcinoid (PC) tumour, a subgroup of pulmonary neuroendocrine tumours (NETs) together with large-cell neuroendocrine carcinoma and small-cell lung cancer [5]. Globally, the incidence of PC tumours varies between 0.5 and 1.5 per 100 000 persons per year [3, 6–8]. PC tumours are further classified by histology into typical carcinoid (TC) and atypical carcinoid (AC) tumours [5]. The primary treatment for PC tumours is surgery [9]. In general, their prognosis is favourable, especially when resected, with a 5-year survival rate of more than 90%, but 10–20% of the tumours metastasize resulting in a lower survival rate [10–13].

Rare cancers are often difficult to study due to a lack of study material. While hundreds of patients may be living with a given rare cancer, the geographic dispersion of the patients limits the number of cases seen at a single institution. Thus, the aim of this study was to collect a nationwide PC tumour cohort through the Finnish biobank infrastructure to study clinical and histological prognostic factors of PC tumours. Moreover, we aimed to explore how the number of tumours found in the Finnish biobanks corresponds to the number of tumours registered by the Finnish Cancer Registry (FCR). We were also interested to compare the biobank database diagnosis, obtained from primary pathology reports, with the diagnosis after re-evaluation of the tumour by an experienced pathologist. Furthermore, we aimed to study the application processing times in biobanks and to identify possible bottlenecks in the sample and data delivery process.

## Materials and methods

### Pulmonary carcinoid incidence data

The FCR operates under the National Institute for Health and Welfare to collect population-based data on the cancer incidence in Finland. Nationwide registration of cancer cases started in 1953 and became compulsory for institutions and health care professionals in 1961 [14]. Disease information is received from public and private hospitals, laboratory reports, and death certificates [15]. The degree of data completeness in cancers affecting the respiratory tract is estimated to be 97.2% [14]. We requested information from the FCR on lung cancer and PC tumour incidence from 1990 to 2013. Age-standardized incidence (per 100 000 person-years) rates were calculated using the world standard population (1966) for weights.

### Collecting a nationwide pulmonary carcinoid patient series

**Fig. 1:**
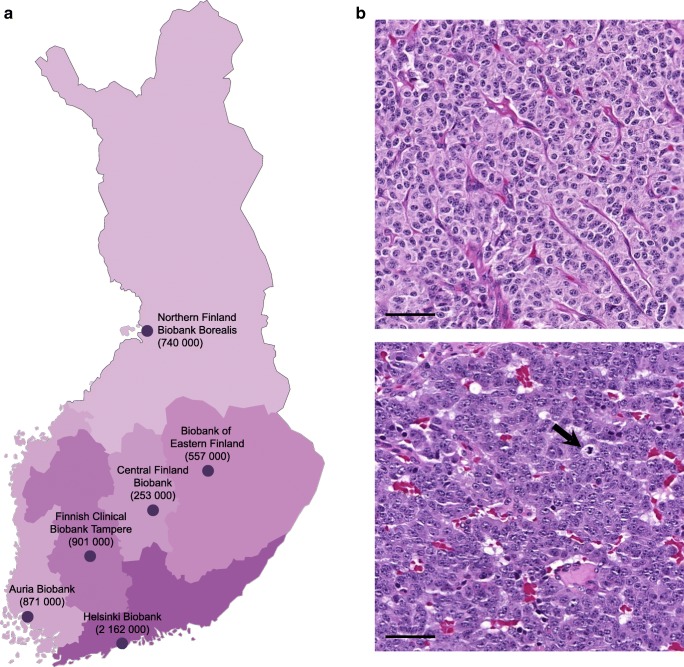
Finnish biobank network and histological images of typical carcinoid (TC) and atypical carcinoid (AC) tumour. **a** All Finnish hospital-integrated biobanks administering pulmonary carcinoid (PC) tumour samples with the population base given in parentheses (as of 31 December 2017). **b** Representative histological images of TC tumour (above) and AC tumour (below, arrow for mitosis). Scale bar 50 μm, original magnification × 40. Images were obtained from digitized slides with the CaseViewer software

Currently, six Finnish hospital-integrated biobanks administer tumour samples of PC patients. These biobanks form a national network covering the whole country (Fig. 1). We applied for primary PC tumour samples resected between 1990 and 2013, coupled with patients’ clinical and outcome data. Biobanks obtained clinical data from hospital registries, survival data from the Population Register Centre, and cause of death data from Statistics Finland.

The Finnish Biobank Act provides a lawful basis for the use of biobanked samples and data for scientific research without project-specific consent from the patients involved. However, each biobank has their own Scientific and Ethical Committee that evaluates the applications and states whether the researchers should be granted access to samples and data. The Scientific and Ethical Committees of all involved hospital-integrated biobanks approved this study.

### Histological re-evaluation of tumour specimens

All histological specimens were re-evaluated from original microscopy slides by a pathologist with special expertise in pulmonary pathology. Tumours were classified according to the World Health Organization 2015 classification criteria, in which TC is defined as presenting < 2 mitoses per 2 mm^2^ and a lack of necrosis whereas AC presents as 2–10 mitoses per 2 mm^2^ and/or foci of necrosis (Fig. 1) [5]. Immunohistochemical labelling against chromogranin A, synaptophysin, and pan keratin were used to confirm the neuroendocrine differentiation.

### Next-generation tissue microarray

The next-generation tissue microarray (TMA) technique relies on careful planning and design, digital pathology, and automated tissue arraying [16]. TMA blocks were created in collaboration with the biobanks. Briefly, after identifying the most suitable formalin-fixed paraffin-embedded (FFPE) tissue blocks per case, fresh slides were sectioned, stained with haematoxylin and eosin, and digitized. Annotations for the TMAs were marked on the digitized slides in accordance with the following principles: two cores from the middle of the tumour, two cores from the tumour border, two cores from the non-tumour area, and one core from the bronchus, if applicable. The TMAs were constructed with TMA Grand Master (3DHISTECH, Budapest, Hungary) or Galileo TMA CK4500 microarrayer (Isenet, Milan, Italy) using 1 mm punches.

### Immunohistochemistry and evaluation of stainings

TMA sections obtained from the biobanks were immunolabelled for chromogranin A, synaptophysin, pan keratin, and Ki-67 in the clinical pathology laboratory at the Helsinki University Hospital as previously described [17]. Briefly, the sections were deparaffinized and antigen retrieval was performed using CC1 reagent (Ventana Medical Systems, Inc., Tucson, AZ, USA) and/or Protease 3 (Ventana Medical Systems). The primary antibodies (chromogranin A (clone DAK-A3, dilution 1:800, Dako, Agilent Pathology Solutions, Santa Clara, CA), synaptophysin (clone SP11, ready-to-use, Ventana Medical Systems), pan keratin (clone AE1/AE3 & PCK26, ready-to-use, Ventana Medical Systems), and Ki-67 (MIB-1, dilution 1:100, Dako) were incubated, and the immunoreactions were detected using either an ultraView or OptiView Universal DAB Detection Kit (Ventana Medical Systems). All slides were counterstained with haematoxylin.

Stained slides were digitized with a Pannoramic scanner (3DHISTECH) and expression patterns for chromogranin A, synaptophysin, and pan keratin were evaluated from the digitized slides as previously described [17]. The proliferation index was evaluated according to the Ki-67 immunoreactivity in the nuclei of the highest labelling region with the ImmunoRatio image analysis software [18].

### Statistical analyses

The Kaplan–Meier method and the log-rank test were applied to estimate cumulative survival probabilities and to graphically display the disease-specific survival (DSS) curves. The log-log 95% confidence intervals (CIs) were calculated for the survival rates. The significance of hazard ratios for clinicopathological variables was tested with univariate Cox regression. Receiver operating characteristics curves were used to estimate a cutoff value for Ki-67 as a prognostic indicator. An arbitrary size of 2.5 cm was used as a tumour size cutoff for survival. Survival was calculated from the date of surgery to the last date of follow-up or death, and non-disease-specific deaths were censored to obtain DSS. A significant difference was predetermined to be a *P* value less than 0.05; two-sided tests were used. Analyses were performed using IBM SPSS Statistics 24 (IBM, Armonk, NY, USA) and SAS for Windows 9.4 (SAS Institute Inc., Cary, NC, USA).

## Results

### Lung cancer and pulmonary carcinoid tumour incidence according to the Finnish Cancer Registry

Between 1990 and 2013, altogether 54 166 lung cancers were registered by the FCR. Of these, 523 (1.0%) were classified as PC tumour. In 1990, the age-standardized lung cancer incidence was 26 per 100 000 persons, and the PC tumour incidence 0.2 per 100 000 persons. By 2013, the lung cancer incidence decreased to 21 per 100 000 persons while the PC tumour incidence increased to 0.34 per 100 000.

### Application processing times in different biobanks

We submitted first two applications for biobanked samples and associated data in October and November 2016. The response times from the approval of the biobanks’ Scientific and Ethical Committees to receiving the samples and data were 11 and 12 months, respectively. At that time, one of the biobanks was not able to deliver electronic medical record information and we had to apply for it from the local university hospital.

The next three applications were submitted in February, May, and July 2017. For these applications, the response times were 15, 17, and 22 months. The sixth application was approved in September 2018 with a response time of 6 months for receiving the samples and data.

### Re-evaluation of the tumours

We obtained altogether 242 tumours, resected between 1990 and 2013, from the biobanks. Of these, seven were excluded due to lack of either chromogranin A or synaptophysin expression, and/or because of a very high Ki-67 proliferation index (> 45%) together with incompatible PC morphology. Three more tumours were excluded because they were metastases from gastroenteropancreatic NETs, not primary pulmonary NETs, and four since only biopsy material was available. In addition, one patient was excluded since he had multiple small tumours and only one was enucleated. Three patients were excluded because of insufficient follow-up data.

The final cohort included 224 tumours. Based on histological re-evaluation according to current diagnostic criteria [5], 21% of the original classifications at the biobank database were changed: 31 TCs were re-classified as ACs and 16 ACs were re-classified as TCs. One tumour diagnosed primarily as neuroendocrine carcinoma was re-classified as TC. In summary, after re-evaluation, 182 (81%) tumours were classified as TCs and 42 (19%) as ACs.

### Comparison between the number of pulmonary carcinoid tumours in the Finnish Cancer Registry and the number of pulmonary carcinoid tumours found in the biobanks

As one of the biobanks was able to provide only tumour samples collected after year 2000, we included in the biobank performance analysis a 10-year period from 2002 until 2011 (Table 1). During this time period, 256 PC tumours were registered by the FCR corresponding to 1.1% of all registered lung cancers (*n* = 23 328). Of these, 233 (91%) were histologically confirmed indicating that these cases might be included in the biobank collections. However, single patients may have been operated on in regional hospitals whose sample archives had not yet been transferred to any biobank.

**Table 1:** Number of pulmonary carcinoid tumours according to the Finnish Cancer Registry (FCR) and the number of tumours found and delivered by the biobanks between 2002 and 2011

University hospital district	Local biobank(s)	FCR all	FCR histologically confirmed	Tumours found in the biobanks		
		(*n*)	(*n*)	orig^a^. (*n*)	deliv.^b^ (*n*)	conf.^c^ (*n*)
Helsinki	Helsinki Biobank	93	85	72	68	66
Turku	Auria Biobank	46	42	42	18	18
Tampere	Finnish Clinical Biobank Tampere	60	54	26	9	7
Kuopio	Biobank of Eastern Finland and Central Finland Biobank	34	30	34	18	14
Oulu	Northern Finland Biobank Borealis	23	22	32	16	13
All		256	233	206	129	117

^a^Number of tumours the biobank was able to identify from its sample registry

^b^Number of tumours the biobank was able to deliver

^c^Number of tumours we confirmed to be primary resected PC tumours based on the morphology, immunohistochemistry, and patient data

Biobanks identified 206 PC tumour tissue samples from their sample registries corresponding to 88% of all histologically confirmed PC tumours registered by the FCR. Of the 206 tumours, biobanks were able to deliver tissue material from 129 (63%) tumours. Of these, we had to exclude 12 tumours (9%) due to reasons stated above. Finally, 117 tumours were included in our patient series corresponding to 57% of all PC tumours identified by the biobanks between 2002 and 2011.

### Demographics and clinical findings of the pulmonary carcinoid patients

Demographic, surgical, histopathological, and clinical characteristics of the patients are described in Table 2. We included here all 224 patients operated on between 1990 and 2013.

**Table 2:** Demographic, histopathological, and treatment characteristics of the patients

Variable	TC		AC		All	
Sex						
Male	66	(36%)	21	(50%)	87	(39%)
Female	116	(64%)	21	(50%)	137	(61%)
Age						
Mean	54		54		54	
Median	55		56		55	
Range	19–86		23–77		19–86	
Location of the tumour						
Right lung	111	(61%)	24	(57%)	135	(60%)
Upper lobe	25		9			
Middle lobe	35		8			
Lower lobe	40		7			
Main bronchus	2		0			
Two lobes	8		0			
Unknown	1		0			
Left lung	69	(38%)	18	(43%)	87	(39%)
Upper lobe	34		7			
Lower lobe	34		6			
Main bronchus	1		3			
Unknown	0		2			
Unknown	2	(1%)	0		2	(1%)
Surgical procedure						
Lobectomy	90	(58%)	24	(60%)	114	(58%)
Sleeve resection	17	(11%)	8	(20%)	25	(13%)
Segmentectomy	17	(11%)	1	(3%)	18	(9%)
Bilobectomy	13	(8%)	2	(5%)	15	(8%)
Wedge resection	12	(8%)	1	(3%)	13	(7%)
Pneumonectomy	3	(2%)	4	(10%)	7	(4%)
Enucleation	3	(2%)	0		3	(2%)
Unknown	27		2		29	
Tumour size (cm)						
≤ 1	46	(25%)	11	(26%)	57	(25%)
1.1–2.5	91	(50%)	19	(45%)	110	(49%)
> 2.5	42	(23%)	11	(26%)	53	(24%)
Unknown	3	(2%)	1	(2%)	4	(2%)
Ki-67 proliferation index						
< 1%	57	(32%)	11	(26%)	68	(31%)
1–2.5%	102	(58%)	21	(50%)	123	(56%)
> 2.5%	18	(10%)	10	(24%)	28	(13%)
Unknown	5		0		5	
Hilar/mediastinal (N1/N2) nodal involvement at diagnosis						
Yes	9	(7%)	8	(21%)	17	(11%)
No	114	(93%)	30	(79%)	144	(89%)
Not examined	59		4		63	
Neoadjuvant treatment						
Chemotherapy	0		1		1	
Radiotherapy	0		1		1	
Both	0		1		1	
Distant metastasis						
At diagnosis	0		2		2	
During follow-up	11		9		20	
Treatment of metastatic disease						
Metastases surgery only	4		0		4	
Chemo/radiotherapy only	1		3		4	
SSA only	2		1		3	
SSA + chemo/radiotherapy	1		4		5	
SSA + PRRT	1		1		2	
SSA + PRRT+chemo/radiotherapy	1		1		2	
No treatment, only follow-up	1		1		2	

*AC* atypical carcinoid, *PRRT* peptide receptor radionuclide therapy, *SSA* somatostatin analogue, *TC* typical carcinoid

Of 224 patients, 161 (72%) underwent lymph node dissection at primary surgery. Two (1%) patients had metastatic lesions in bones, liver, and pleura at the time of primary surgery. In addition, during the follow-up, 20 (9%) patients developed a recurrent disease and/or distant metastases in bones, liver, adrenal gland, brain, ovary, or pancreas. The average time from primary surgery to the recurrent or metastatic disease was 58 months (range 3–224 months).

### Survival analysis

The median follow-up time of the patients was 11.4 years (mean 12.7; range 0–28.0 years). Of 224 patients, 47 died during the follow-up. Fourteen patients died with evidence of recurrent or metastatic carcinoid tumour (6 TC patients and 8 AC patients), 2 patients died because of complications after surgery, and 31 patients died from unrelated causes. The average survival time for the patients with disease-specific death was 6.8 years (range 1.1–17.4 years).

The disease-specific 5- and 10-year survival rates among all PC patients were 97% (95% CI, 94–99%) and 95% (95% CI, 91–97%), respectively. Among the TC patients, 5- and 10-year DSS rates were 99% (95% CI, 96–100%) and 98% (95% CI, 94–99%), respectively. Among the AC patients, the 5-year DSS rate was 90% (95% CI, 76–96%), and the 10-year DSS rate 81% (95% CI, 63–90%).

**Fig. 2:**
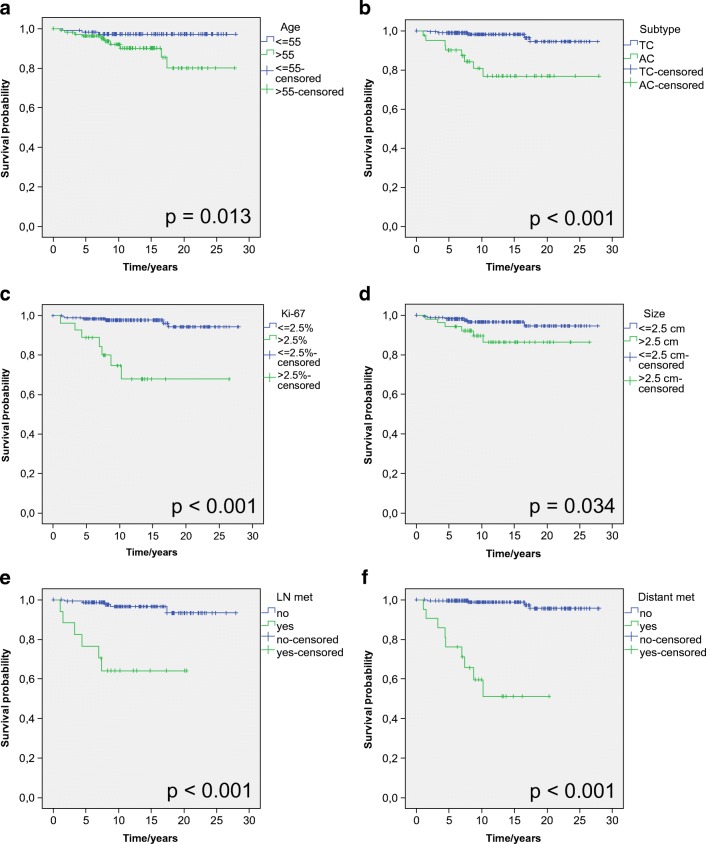
Disease-specific survival probabilities based on age at the time of surgery (**a**), histological subtype (**b**), Ki-67 proliferation index (**c**), tumour size (**d**), presence of lymph node involvement at the time of primary surgery (**e**), and presence of metastatic disease during follow-up (**f**). *P* values were calculated with the log-rank test. AC, atypical carcinoid; LN, lymph node; TC, typical carcinoid

We evaluated the association between clinical and pathological variables and patient outcomes with the Kaplan–Meier method and the log-rank test. We found that age over 56 years at the time of diagnosis, tumour size over 2.5 cm, atypical histology, Ki-67 proliferation index higher than 2.5%, hilar/mediastinal lymph node involvement at the time of diagnosis, and the presence of metastatic disease were associated with a shorter DSS (Fig. 2). In univariate Cox survival regression analysis, all abovementioned factors were associated with a risk of worse outcome (Table 3). Because of a low number of disease-specific deaths (*n* = 14), we could not perform a reliable multivariate analysis.

**Table 3:** Analysis of potential risk factors for disease-specific mortality using univariate Cox regression

Risk factor	*p* value	HR (95% CI)
Age (> 56 vs. ≤ 55)	0.023	4.5 (1.2–16.7)
Gender (female vs. male)	0.281	0.5 (0.2–1.6)
Tumour size (≥ 2.5 cm vs < 2.5 cm)	0.045	3.2 (1.0–9.9)
Histological type (AC vs. TC)	< 0.001	7.8 (2.5–23.8)
Ki-67 proliferation index (≥ 2.5 vs < 2.5)	< 0.001	11.518 (3.7–35.8)
Hilar/mediastinal lymph node involvement at the time of primary surgery (yes vs. no)	< 0.001	11.9 (3.6–39.2)
Presence of metastatic disease (yes vs. no)	< 0.001	34.7 (10.1–119.8)

*AC* atypical carcinoid, *CI* confidence interval, *HR* hazard ratio, *TC* typical carcinoid

## Discussion

In the present study, we collected one of the largest PC tumour patient series reported, utilizing the Finnish biobank infrastructure. In this series of 224 tumours, we identified six factors that seem to predict PC patient outcome: age over 56 years at the time of diagnosis, tumour size over 2.5 cm, atypical histology, Ki-67 proliferation index higher than 2.5%, hilar/mediastinal lymph node involvement at the time of diagnosis, and the presence of metastatic disease. We also showed that a nationwide hospital-integrated biobank network makes it possible to collect reasonably large cohorts of rare diseases.

Between 2002 and 2011, 251 PC tumours were registered by the FCR, corresponding to 1.1% of all lung cancers in Finland. This percentage is equal to reports from other Nordic countries [19, 20]. However, the proportion of AC tumours (*n* = 10, 4%) was low. This is mainly explained by the nomenclature applied by the FCR: ICD-O-3 was introduced in 2007, and ACs have been registered as ACs only after that. Before 2007, ACs were registered as neuroendocrine NOS. Additionally, we observed an increase in PC tumour incidence from 1990 to 2013. The same has been noticed in previous reports [6, 21, 22]. Possible explanations include improved imaging methods and diagnostic protocols together with better awareness among clinicians.

The six Finnish biobanks included more than 88% of the cases registered by the FCR. The rest of the samples are probably stored in smaller pathology departments that have not yet transferred their sample repositories to any biobank. However, biobanks were able to deliver only 63% of their found samples to us. One main reason was paucity or lack of tissue material. According to the Finnish Biobank Act, a sample collected primarily for diagnostic purposes can be used for research purposes only if possible future patient care is not jeopardized. This dualistic nature of biobanked samples prevents delivering scarce samples for research. In addition, the preliminary search for tumour numbers is based on the registry information the biobank received from the pathology laboratory at the time the samples were transferred into the biobank. In some cases, the primary diagnosis in the database was incorrect and the biobank pathologist scanning the cases excluded these tumours (e.g. paragangliomas). Also, a minority of the samples found were biopsies unsuitable for our study. Moreover, several samples were not found in the biobank archives. A plausible explanation for this is the practice before the biobanking legislation: the use of samples for research purposes was not registered and followed-up diligently, and some samples could thus not be located anymore.

Based on our experience, the sample application process was slow in all biobanks. One obvious reason was that for most of the biobanks, our application was among the first ones, and the sample and data delivery processes were still being built. This concerns especially the collection of clinical follow-up data from the patient records. Biobanks also faced challenges in finding patient data for older cases since, based on the Decree of the Ministry of Social Affairs and Health on Patient Records (298/2009), Finnish hospitals are regulated to conserve the patient data for only 12 years after the patient’s death. Also, even though patient data has been collected in electronic form for almost two decades, it is mainly not in a structured form but as freely written text. Free text, together with several different software programs applied nationwide for storing it, places a major challenge for mining patient data. Understandably, in addition to technical skills, medical knowledge is needed for accurate patient data mining. We noticed clear regional differences in the capability of the biobanks in collecting and processing the patient data.

One special feature of the Finnish biobanking is the possibility to combine patient data with national registry data. Since 1964, all Finnish citizens have had a unique personal identification number that follows the person from birth to death [23]. In addition, record keeping in general for administrative and statistical purposes has a long tradition in Finland. Due to these two issues, combining patient data from local hospital records with national records is straightforward. For this study, the biobanks obtained the patient survival data from the Population Register Centre and the cause of death data from Statistics Finland.

All Finnish biobanks have excellent facilities and technical skills for processing FFPE tissue into TMA format. The samples were digitized, which allows the researchers to choose the morphologically most suitable samples for their study as well as to annotate representative areas for TMAs by themselves, despite the geographic location. In general, two copies of the TMA blocks are prepared and the biobanks deliver researchers unstained sections, not the TMA blocks. This allows the TMA material to be used in other studies in the future as well.

In addition to general slowness of the biobanks’ processes, a major bottleneck in the sample request process was submitting separate applications to each biobank, with varying forms and content. However, since the beginning of our study in 2016, the situation has improved. Today, all hospital-integrated biobanks can be approached with one application through the Fingenious gateway (www.fingenious.fi, The Finnish Biobank Cooperative).

Regardless of the challenges, we succeeded in collecting a nationwide PC tumour patient series coupled with clinical follow-up and outcome data. Histological re-evaluation of the tumours resulted in changed classification in 21% of the tumours. Based on the TMA sections and clinical information received from the biobanks, we performed survival analysis. The 5- and 10-year DSS rates were higher than in most previous studies [13, 24, 25]. The main reason for this might be that we calculated the DSS while others reported the overall survival.

As expected and shown earlier, atypical histology, hilar/mediastinal lymph node involvement at the time of diagnosis, and the presence of metastatic disease are associated with a worse outcome [10, 12, 13, 17, 26]. Previous reports have also pointed out the importance of the Ki-67 proliferation index as a prognostic factor [27–30]. Tumour size as well as age at the time of diagnosis are identified as prognostic factors with different cutoff values [17, 26, 31, 32].

The data on PC tumours presented here together with the stained TMA sections are now available to other researchers on application. The Finnish Biobank Act enables the use of samples and data for both academic and industrial research purposes, both in Finland and abroad. In addition, the study results should enrich the biobank through the return of raw data from the researchers’ experiments to the biobank.

In conclusion, the Finnish biobank infrastructure offers excellent opportunities for medical tissue-based research. Biobanks were able to find 88% of the patient cases registered in the nationwide FCR and process 63% of them into TMA format. The main bottleneck in the process was collecting clinical follow-up data because of insufficient medical and technical expertise in the biobanks. To be able to develop the biobank process further, involving more medical knowledge in the sample and data acquisition is a necessity. Also, when working with tissue samples collected over decades, pathology expertise is needed for histological re-evaluation of the samples.
